# Effects of probiotic and synbiotic supplementation on ponderal and linear growth in severely malnourished young infants in a randomized clinical trial

**DOI:** 10.1038/s41598-023-29095-w

**Published:** 2023-02-01

**Authors:** Sharika Nuzhat, S. M. Tafsir Hasan, Parag Palit, Md Ridwan Islam, Mustafa Mahfuz, M. Munirul Islam, Md Ashraful Alam, Robin L. Flannery, David J. Kyle, Shafiqul A. Sarker, Tahmeed Ahmed

**Affiliations:** 1 Nutrition and Clinical Services Division, International Centre for Diarrhoeal Disease Research, Bangladesh (icddr,b), 68, Shaheed Tajuddin Ahmed Sarani, Mohakhali, Dhaka 1212 Bangladesh; 2 Evolve BioSystems, Inc., Davis, CA 95618 USA

**Keywords:** Epidemiology, Paediatric research, Clinical trial design

## Abstract

Severe acute malnutrition (SAM) is a major global public health problem. We aimed to assess the effects of probiotic and synbiotic supplementation on rate of weight gain and change in length in young SAM infants. This study was substudy of a single-blind randomized clinical trial (NCT0366657). During nutritional rehabilitation, 67 <6 months old SAM infants were enrolled and randomized to receive either probiotic (*Bifidobacterium. infantis* EVC001) or synbiotic (*B. infantis* EVC001 + Lacto-N-neotetraose [LNnT]) or placebo (Lactose) for four weeks and were followed for four more weeks after supplementation. In multivariable linear regression model, the mean rate of weight gain in the probiotic arm compared to placebo was higher by 2.03 unit (P < 0.001), and 1.13 unit (P = 0.030) in the synbiotic arm. In linear mixed-effects model, mean WAZ was higher by 0.57 unit (P = 0.018) in probiotic arm compared to placebo. Although not statistically significant, delta length for age z score (LAZ) trended to be higher among children in probiotc (β = 0.25) and synbiotic (β = 0.26) arms compared to placebo in multivariable linear regression model. Our study describes that young SAM infants had a higher rate of weight gain when supplemented with probiotic alone, compared to their counterparts with either synbiotic or placebo.

## Introduction

Globally, 13.6 million children under the age of five are affected by severe acute malnutrition (SAM)^1^, the most severe form of undernutrition. Malnutrition is responsible for around 60% of childhood deaths under the age of 5 years and two-thirds of those occur during 1st year of life^2^. Despite introducing standardized treatment and re-feeding protocols, inpatient mortality reaches up to 30% in many hospitals^3–6^. Bangladesh also contributes to this high inpatient mortality rate owing to the high prevalence of malnutrition in the country^7^. The situation is further deteriorating as the COVID-19 pandemic is having a devastating impact on food security^8^. Infancy represents a critical window when the gastrointestinal system develops rapidly, a process that accompanies characteristic age-specific changes in the intestinal microbiota^9–11^. Recent reports suggest about four million infants aged under 6 months are suffering from SAM worldwide^12,13^. The mortality of children with SAM is primarily associated with diarrhea, intestinal inflammation, and systemic inflammation^14^. However, these associations are not solely mediated by the presence of enteropathogens^15^, and metagenomic and molecular approaches have revealed substantial loss of microbiome diversity in large gut of children with SAM^16^.

The human digestive system possesses a diverse microbial community (the gut microbiome) which mediates various metabolic functions essential to the host physiology^17^. Probiotics are live, commercially-produced bacterial taxa that are generally recognized as safe (GRAS) when administered in appropriate levels to the host^18^. Among the most common probiotics are various species and strains within the genera, *Lactobacillus* and *Bifidobacterium*^19–24^. *Bifidobacterium* sp. constitutes the most abundant member of the gut microbiota in young infants. The nutritional value of foods is influenced partially by the intestinal microbial community and the and the ability of that community to break down dietary oligosaccharides (fibre components) that are not digestible by the host^25^. Evidence suggests that children’s nutritional status is also connected to their gut microbial community^26^. Recent works indicate that children with SAM have been found to suffer from a condition known as gut dysbiosis or the imbalance in the microbial community, which subsequently mediates some of their nutritional deficiencies and pathological conditions^16,27–31^.

Prebiotics generally refer to oligosaccharides that are indigestible to the host, but are consumed by gut microflora, including any added probiotics^32^. These undigested dietary carbohydrates are fermented by colonic bacteria in order to produce short-chain fatty acids (SCFA) as end products that can provide nourishment to the colonic cells^33^, lower the colonic pH, minimize enteropathogen growth, and support the development of the immune system. Human milk contains an abundance of unique prebiotic oligosaccharides, known as human milk oligosaccharides (HMOs) which are primarily metabolized by infant-specific gut microbiotia such as *Bifidobacterium infantis*^34^ producing acetate and lactate as end products. Infants who are not breast fed have a paucity of *B. infantis* in their gut microbiome which gives rise to a more diverse gut microbiota containing elevated levels of opportunistic pathogens due to the deficiency of HMO^35^. Ready-to-Use Therapeutic Foods (RUTF) are used today to facilitate weight gain in millions of children threatened by acute malnutrition in the world today. However, such foods do not contain any HMO for the support of infant-spedific gut bacteria and the effort and time required to feed an infant several times per day may not be feasible for mothers of low socioeconomic status as they are involved in different types of employment and activities^36^. To improve the rate of weight gain in severely malnourished children and prevent malnutrition, different interventions are being carried out in different countries, for example: l-carnitine supplementation^37^, Lipid-Based Nutrient Supplements^38^, ready to use therapeutic foods^39^ etc. Several researchers have reported a positive association of probiotic supplementation with child growth^40^. However, none of these studies were conducted in low-and-middle-income settings involving young infants aged less than 6 months^40^ and these studies vary widely in the probiotic taxa used, most of which do not carry HMO processing genes. Decreased breastfeeding practices have been reported among children with severe acute malnutrition^41^, implying reduced availability of prebiotic human milk oligosaccharides (HMOs), which are in turn needed to promote the growth of age-specific probiotic microbiota, such as *B. infantis*^42^. A recent systematic review has reported that probiotics have a heterogeneous effect on linear growth of healthy children in low and middle income countries^43^.

This current study was conducted to explore the role of probiotic and synbiotic supplementation on the ponderal and linear growth of partially/non-breastfed infants of 2–6 months of age with SAM, recovering from acute illness in a hospital.

## Methods

### Study design

It was a single-blind randomized clinical trial.

### Study settings and participants

The study was conducted at Dhaka Hospital (icddr,b). The institution has well-equipped clinical care set ups as well as laboratory facilities capable of performing all the analyses required. After acute phase management, children with severe acute malnutrition (SAM) were transferred to the Nutritional Rehabilitation Unit (NRU) for stabilization and preparation for discharge for home.

### Working definitions

#### Severe acute malnutrition (SAM)

Defined by weight for length Z score < − 3, according to WHO growth standard or presence of bilateral pedal edema, independent of anthropometric measurements^44^.

#### Study completion of an infant

From the start of enrolment, 28 days supplementation period, and four weeks of post supplementation follow-up.

#### Rate of weight gain

The rate of weight gain (gm/kg/day) was measured using the following formula,

$$Rate\,of\,weight\,gain=\frac{Weight\,on\,discharge-Weight\,on\,study\,completion\,(gm)}{Total\,duration\,of\,days\,between\,discharge\,and\,study\,completion*Weight\,on\,discharge\,in\,kg}$$

### Trial groups with interventions

SAM infants between 2 and 6 months of age were randomized upon completion of antibiotic treatment at the NRU into one of the three supplementation groups:

- Placebo (Lactose)
- Probiotic (*B. infantis* EVC001)
- Synbiotic: Probiotic (*B. infantis* EVC001, 8 billion CFU/day) + prebiotic (Lacto-N-neotetraose -LNnT)

Each group was assigned to receive the standard of care and diet as practiced in Dhaka Hospital, icddr,b.

### Randomization

Randomization for supplementation allocation was done in a 1:1 ratio using stratification by age (2–3.9 months vs. 4–5.9 months) to ensure equal participation of infants by age groups. Allocation was achieved by using random permuted blocks of the size of three prepared by a third party not related to the study.

### Sample size

For this pilot trial, the sample size was 67 SAM infants. As it was a pilot trial, the minimum number of patients was included in each arm.

### Ethical approval

The randomized controlled trial was registered at ClinicalTrials.gov (NCT03666572) on 12/09/2018 (https://clinicaltrials.gov/ct2/show/NCT03666572) and the research protocol was approved by the Institutional Review Board of icddr,b (Protocol reference no. PR-17112) which is an independent body that approves the research protocol, Secures the rights of the participants and investigators. Written informed consents were obtained from the legal guardians or parents of the participants prior to their inclusion in the study. All procedures followed in the present study were in accordance with the ethical standards of the responsible committee on human experimentation (institutional and regional) and with the Helsinki Declaration of 1964. It is registered with the ClinicalTrials.gov and the registration number is NCT03666572. All clinical aspects of the study were supervised by the investigators and study physicians. All activities were conducted in accordance with the Helsinki Declaration of 1975, revised in 1983. Names of the participants were de-identified prior to analysis.

## Management of the study participants

### SAM infants

Eligible SAM infants were treated for the acute phase management in the Longer Stay Unit by the standardized icddr,b treatment protocol for children with SAM^45^. Infants who recovered from acute illness and stabilized were transferred to the NRU and randomized and enrolled into the treatment groups by study physicians. Caregivers of the infants were blinded after assignment to supplements. In the acute phase, infants were managed with therapeutic milk (F-75; WHO protocol). In the NRU, F-75 was replaced by F-100 therapeutic milk (WHO protocol)^46^. The nutrient composition of both diets are included in the supplementary table. Both the feeds were prepared by the hospital dietician, and F-100 was provided with a frequency of every 2–3 h, and a daily volume of 150 ml/kg for non-edematous infants and 130 ml/kg for edematous infants. In addition to feeding as a routine practice, all caregivers at NRU received sessions on psychosocial stimulation, immunization, follow-up, etc. Breastfeeding was encouraged in between the feeds. The amount of breast milk consumed was measured by the test weighing method^47^. The contents of sachets of placebo or probiotic were mixed with a teaspoon of infant formula or breast milk and fed to the infant once a day. The prebiotic, LnNT, was mixed with F-100 (1.6 g in 200 ml of F-100) during the hospital stay.

The infants were discharged upon being declared clinically well and, if possible, upon attainment of a WLZ ≥ − 2^48^. The caregivers had been trained by study personnel on the proper administration of the supplements and reconstitution of infant formula. After discharge from the hospital, the caregivers of the infants in the synbiotic arm were advised to mix one sachet of prebiotic (1.6 g) with 120 ml of infant formula to be given twice a day to complete the total duration of supplementation of 4 weeks.

Infant formula provided to the study participant mothers who were not breastfeeding or patially breast feeding their infants did not contain any probiotics or prebiotics.

### Follow-up

Infants were followed up through home visits by Field Research Assistants (FRA) twice weekly throughout the entire study period of 8 weeks (4 weeks supplementation + 4 weeks post-supplementation follow-up). Caregivers were asked to keep empty sachets after administration of supplements. FRAs collected the information on daily administration of supplements. Missing doses were not repeated. Infants developing an infection during the follow-up period were re-admitted to the hospital.

### Outcome measures

- Change in rate of weight gain (g/kg per day) with supplements
- Change in Length-for-age Z score with supplements

### Data collection

Data were collected by study physician in the case record forms. Data were randomly checked by co-investigators. Data will be immediately copied on the hard disks of two computers as soon as data verification is completed.

### Data analysis

Data were analyzed through the statistical software Stata (15.0 version for windows) after cleaning with repeated checks. To explore demographic, clinical, and socioeconomic data, we used the median with an interquartile range (IQR) for asymmetric quantitative data and frequency with proportion for categorical data. To evaluate the difference in baseline characteristics Krusker wallis test was done in the continuous dataset of three supplements. Chi square test was done for categorical data set of baseline characteristics.

As 47 infants had edema during enrolment, the rate of weight gain was calculated from the change of weight at hospital discharge to the end of study period. It was not possible to calculate WAZ during enrolment of patients with nutritional edema as it would not represent their actual Z score. To keep the uniformity of data, the change in LAZ was assessed from discharge to study completion.

The effect of the supplements on the rate of weight gain of the infants as well as the change in LAZ from discharge to study completion was examined using multivariable linear regression models. The rate of weight gain model was adjusted for sex, age (in months), and WAZ at the time of discharge. The change in LAZ model was adjusted for sex, age (in months), and LAZ at the time of discharge. For a sensitivity analysis, we examined the effects of probiotic and synbiotic supplementation on weekly WAZ scores from the first week following enrollment to study completion in a mixed-effects multiple linear regression model specifying a random effect at the child level to account for within-child correlations. The mixed-effects model was adjusted for sex, age (in months), and WAZ at enrollment. We had 8 data points per infant in our total 8-week study period for WAZ. Three infants had edema in the first week following enrollment, so they were excluded and analysis were carried out on 64 infants. The strength of association was expressed as β (95% CI), where P < 0.05 was considered significant.

## Results

A total of 67 SAM infants were included in the analysis. Out of 67 SAM infants, 23 infants were in the placebo group, 21 infants were in the probiotic group, and 23 infants were in the synbiotic group. Table 1 demonstrates the sociodemographic, clinical, and anthropometric characteristics of the three groups. The median age of infants in placebo, probiotic and symbiotic groups were 120, 119, and 120 days respectively. The median birth weight of infants was 2.6 kg in placebo, 2.65 kg in probiotic, and 2.8 kg in the synbiotic group. The median gestational age was 37 weeks (calculated from mothers’ history of pregnancy) among all the infants in the three groups. Out of 67 infants, 47 (70%) of the infants were edematous. Only 31 infants were breastfed, and the median percentage of nutrition received from breast milk was generally low in all groups (4.92% (placebo) vs. 6.42% (probiotic) vs. 00% (synbiotic)). The median duration of hospital stay was 11 days with placebo 12.5 days with probiotic 11 days with synbiotic groups. All the baseline characteristics of three treatment arms were comparable.

**Table 1:** Sociodemographic, anthropometric and clinical characteristics of SAM infants of different supplementation arms on admission.

Characteristics	Placebo (n = 23)	Probiotic (n = 21)	Synbiotic (n = 23)	P value
Age in days, (median, IQR)	120 (52)	119 (42)	120 (68)	0.686
Sex: male, n (%)	13 (56.5%)	12 (57.14%)	13 (56.52%)	0.999
Gestational age (weeks), (median, IQR)	38 (4)	38 (3)	36 (3)	0.166
Delivery by C-section, n (%)	11 (33.33)	14 (42.42)	8 (24.24)	0.106
Birth weight (kg), (median, IQR)	2.6 (1.1)	2.65 (0.65)	2.8 (1)	0.467
Mother’s age (year), (median, IQR)	23 (12)	23 (6)	23 (7)	0.735
Maternal education < 5 years, n (%)	10 (43.48%)	9 (42.86%)	10 (43.48%)	0.999
Housewife mother, n (%)	15 (65.22%)	16 (76.19%)	20 (86.96%)	0.224
Family income (taka), (median, IQR)	15,000 (10,000)	15,000 (6000)	15,000 (13,000)	0.837
Body weight on admission (kg), (median, IQR)	4.59 (1.13)	4.52 (0.53)	5 (1.24)	0.357
Length on admission (cm), (median, IQR)	58.5 (6)	57.6 (3)	59 (6.5)	0.559
Admission LAZ (median, IQR)	− 2.23 (1.41)	− 1.62 (1.6)	− 1.43 (1.99)	0.255
MUAC (median, IQR)	120 (12)	115 (15)	120 (8)	0.615
Presence of bilateral pedal edema (%)	16 (69.6%)	13 (61.90%)	18 (78.26%)	0.495
Duration of diarrhea, days (median, IQR)	3 (3)	2 (2)	3 (3)	0.925
Presence of cough on admission, n (%)	2 (8.70%)	2 (9.52%)	4 (17.39%)	0.607
Percentage of breast milk intake (median, IQR)	4.92 (25.9)	6.42 (24.83)	0.00 (11.33)	0.170
Duration of hospitalization, days, (median, IQR)	11 (5)	11 (14)	11 (6)	0.659

Table 2 shows the rate of weight gain among the different supplementation groups from discharge to study completion. The mean rate of weight gain was highest in probiotic arm (5.90 g/kg/day) among all three supplementation groups.

**Table 2:** Rate of weight gain for SAM infants with three supplementations (from discharge to study completion).

Study arms	Rate of weight gain (gm/kg/day), mean (95% CI)
Placebo (n = 23)	4.35 (3.40, 5.30)
Probiotic (n = 21)	5.90 (4.70, 7.09)
Synbitotic (n = 23)	4.77 (3.67, 5.87)

In the adjusted model, a significant increase in weight gain of 2.03 units was observed with the probiotic group compared to the placebo group. A lesser, but still significant increase in weight gain vs. placebo was also seen in the symbiotic group (Table 3). On the other hand, the the probiotic or symbiotic use appeared to have no significant impact on LAZ from discharge to study completion after adjusting for age during discharge from hospital, sex, LAZ during discharge from hospital (Table 4).

**Table 3:** Infant weight gain from discharge to study completion in Probiotic and Synbiotic Groups vs, placebo after adjustment for age during discharge from hospital, sex, WAZ during discharge from hospital.

	β	95% CI	P value
Probiotic	2.03	1.00, 3.06	< 0.001
Synbiotic	1.13	0.11, 2.15	0.030

**Table 4:** Change in infant length for age (LAZ) from discharge to study completion in Probiotic and Synbiotic Groups vs, placebo after adjustment for age during discharge from hospital, sex, LAZ during discharge from hospital.

	β	95% CI	P value
Probiotic	0.25	− 0.08, 0.59	0.130
Synbiotic	0.26	− 0.07, 0.59	0.116

In the linear mixed-effect model the probiotic was significantly associated with the weekly change in WAZ after for adjusting sex, age and WAZ during the first week Table 5). In the adjusted model, a statistically significant weekly change in WAZ of 0.57 units was found in the probiotic supplement.

**Table 5:** Linear mixed-effects models to identify the longitudinal effects of probiotic or synbiotic on WAZ (from the first week following enrollment to study completion).

	β	95% CI	P value
Probiotic	0.57	0.10, 1.04	0.018
Synbiotic	0.14	− 0.39, 0.66	0.613

Placebo is the reference group. Adjusted for age on 1st week, sex, WAZ on 1^st^ week.

## Discussion

There is paucity of studies assessing the impact of probiotic and synbiotic supplementation on rate of weight gain among young malnourished infants. In this respect, this is the first randomized clinical trial on probiotic and synbiotic supplementation in young infants with severe acute malnutrition. The most important finding of this study was that the infants supplemented with *B. infantis* EVC001 (the probiotic group) demonstrated better weight gain in comparison to the other two groups who were supplemeted with either the synbiotic or placebo.

The mean rate of weight gain for SAM infants (discharge from hospital to study completion) was 4.35 g/kg/day in placebo arm, 5.90 g/kg/day in probiotic arm and 4.77 g/kg/day in synbiotic arm, with the rate for the probiotic arm being graded as moderate by WHO^49^. All the infants included in the study received standardized management of malnutrition. Their diet was also similar, F-75 followed by F-100 and at home infant formula for those who were partially or non-breastfed. The surprisingly reduced growth rate of the synbiotic group relative to the probiotic group may be a consequence of the complete lack of breast milk nutrition in the in the symbiotic group vs. a median of 6.4% of calories from breast milk in the probiotic group (Table 1). The range of HMO found in the small amount of breast milk may have had a greater impact on proliferation of *B. infantis* and subsequently on the health and growth of the infant than the small amount to one specific HMO (LnNT) provided in the symbiotic group. synbiotic group. HMOs modulate health by serving as decoy receptors for many opportunistic infectious agents^50^, improves host defence mechanism by strengthening the gut barrier activities^51^, and improves immunity.

More complex HMOs, LNT, and LNnT core can comprise up to 70% of total HMOs^52^. *B. infantis* strains characterized to date can grow in vitro with HMOs as a sole carbon source; monosaccharides from HMOs are key components for the “bifid shunt” (fructose-6-phosphate phosphoketolase pathway), which produces ATP, acetate, and lactate as end products^53^. With this special metabolic process *B. infantis* is able to utilize LNT and LNnT (with Bifid shunt)^54^. In addition safety of LNnT was evaluated by EFSA (European Food Safety Authority) for infants^55^. For these reasons, we used LNnT as prebiotic in our study with *B. infantis*, a known consumer of prebiotic.

Follow-up after discharge from NRU can help to assess the growth and development of children. Previous studies have highlighted the mortality outcome after discharge from facility-based management of SAM^56,57^. However, the rate of weight gain was not calculated in any of these studies. During recovery phase many studies have shown rapid weight gain^58^ and during this period proper energy intake is important. Recently, researchers have proposed at least three possibilities for this change in growth rate: (1) a change in total nutritional intake (i.e., food composition and amount); (2) more efficient utilization of protein and calories throughout the period of rapid growth; and (3) differences in the composition of the tissue being laid down^59^. In absence of any other supplementary food consumption relative to the placebo control group, we found a weight gain of 5.90 g/kg/day in the probiotic group after discharge from hospital to the end of study. The rate of weight gain observed in this trial by simply providing a specific infant-oriented probiotic (*B. infantis* EVC001) with no other nutritional changes was comparatively higher than that found in other studies with supplementary foods^60^.

The importance of the gut microbiome in child growth has been well recognized in previous studies. Some probiotics may help to improve child growth indirectly through the prevention of infections and micronutrient and vitamin deficiencies (e.g., calcium, zinc and vitamin B12)^61,62^, and/or potentially decreasing the risk of anaemia^63^_._

The effect of a four-probiotic supplement (Synbiotic 2000 Forte) comprising *Pediococcus pentoseceus, Leuconostoc mesenteroides*, *Lactobacillus paracasei, and Lactobacillus plantarum* on the growth of under-nourished children in a large RCT (PRONUT study) was studied by Kerac et al*.*^64^. In that trial, probiotics did not appear to give any benefits on the health or the nutritional condition of these children^64^. Importantly, the probiotics used in the PRONUT trial represent bacteria not commonly found in any large numbers the human gut and especially the infant human gut. Furthermore, the age range of the subjects in the PRONUT trial was from 5 months to 14 years, the children were HIV positive, and being treated with antibiotics^65^, which would have had a significant impact on the gut microbime community itself.

The benefit of dietary intake of probiotics mixed with infant formula in terms of weight and height gain in malnourished children and possible benefits in terms of weight gain in well-nourished children in developing countries has been reviewed^65^. However, results were inconsistant as the studies varied in type and quantity of probiotic taxa given, duration of interventions, characteristics of participants, setting, and units of outcome measures. Several studies were conducted in different countries to determine the anthropometric changes with probiotic supplementation but these were limited to children less than 5 years of age^61,63,66,67^.

Dietary carbohydrates, especially the fibre that is not digested in the upper gastrointestinal system, are known to boost growth and functionality of the gut microbiota community^68,69^. Use of specific prebiotics as dietary fibers has been shown to stimulate satiety hormones and improve appetite control, which may assist in body weight control^70^. Consequently, some researchers have focused on the synergistic effect of pre- and probiotic combinations and the modulation of the immune system by combining probiotics with LC-PUFA (long chain polyunsaturated fatty acids)^64,71^. Other studies in the review, also added either prebiotics or LC-PUFA did not explain any major benefits in developed country settings^72,73^.

We have observed that probiotic supplements comprising *B. infantis* EVC001 result in a better rate of weight gain in comparison to the synbiotic supplementation. In addition, the weekly change of WAZ also shows better result when predicted by the probiotic supplement. The trend in the change of LAZ, from hospital discharge to study completion is also comparately better in the symbiotic supplementation arm (0.26 unit). The small sample size and short duration of follow up, might have an impact on insignificant difference in LAZ.. However, a study in Kenya reported that no significant benefit in height for age z score^74^ could be achieved in spite of inpatient medical and nutritional management for complicated SAM followed by outpatient therapeutic feeding and active follow-up to 1 year with access to additional care and consultant services. In that study, a rapid weight gain was nevertheless observed. In the limited period of 1 month, we were unable to find any significant change in linear growth among the SAM infants supplemented with the probiotic/synbiotic.

A systematic review has shown the effect of different probiotics in different studies^65^. In our study we have focused on supplementation of the infants with only *Bifidobacterium infantis* which is the predominant bacteria during infancy and is subsequently depleted among SAM infants resulting to persistent immaturity of gut microbiota^16^. The recently published SYNERGIE trial observed that *B. infantis* EVC001 supplementation can improve weight gain and reduce intestinal inflammation in young severely malnourished infants^75^. The SYNERGIE trial also reported that a extant strain of *Bifidobacterium infantis* (*B. infantis* Bg_2D9) derived from a healthy Bangladeshi infant contained most of the genes associated with the metabolism of human milk oligosaccharides found in the EVC001 strain of this study, but was missing key HMO transporters located on the H5 gene cluster (Blon 2175–2177). However, when this *B. infantis* Bg_2D9 was introduced to mice models raised in gnotobiotic environment and colonized with fecal microbiota of SAM infants, and fed a Mirapur diet, increased weight gain over the EVC001 strain was observed^75^. This was likely because the Bg_2D9 strain also contained glycosyl hydrolases that were able to deconstruct the plant fiber found in the Mirapur diet but not found in breast milk suggesting the Bg_2D9 strain may be most effective for weaning or transitional diets whereas the EVC001 strain would be preferred for infants getting most of their nutrition from human milk. The gnotobiotic mouse model data with Bg_2D9 may or may not be predictive of weight gain, weekly change of WAZ, Delta LAZ of the infants with supplementations in this study.

We have observed that approximately 15% of the study infants with SAM (n = 9) became sick and were shifted to the longer stay ward while on supplementation in NRU and then treated accordingly. When they became well and were discharged from hospital they continued with the study supplements at home. The reasons of transfer from the NRU were pneumonia (6 cases), diarrhea (2 cases) and sepsis (1 case). These infections were thought to be hospital acquired and the rate was similar to a previous study conducted in same setting^76^. Some studies demonstrated that relapse of SAM or poor weight gain in spite of protocolized management could be related to anthropometric indices on admission^77,78^.

One limitation of the study was that the infants were only followed for a period of 8 weeks so longer term outcome effects of the supplements are unknown. We had no record on the volume of breast milk or infant formula during follow up period. We could not control the use of antibiotics during follow up period in the community as they may have visited local physicians or pharmaciess without informing the study staff. Although our research assistants collected data twice a week from SAM infants the actual intake of feed was not meticulously measured and we had to rely on the mothers’ information on diet and supplemetation. Daily visit of field staffs could minize these undue activities of caregivers. Finally, some of the infants started complementary feeding after 6 months and such feeding data were not included in the analysis.

The findings from the study support the possibility of establishing a moderate weight gain in infants hospitalized with SAM by using a probiotic supplementation protocol comprising *B. infantis* in the post-SAM period without any further changes in their diet. A larger trial with a longer follow up period is required to assess the long term effects of probiotic supplementation on ponderal as well as linear growth of infants with severe malnutrition.

## Supplementary Information

Supplementary Information.

## Data Availability

This data set contains some personal information of the study patients (such as name, admission date, month, area of residence). Thus, the policy of our center (icddr,b) is that we should not make the availability of whole data set in the manuscript, the supplemental files, or a public repository. Our IRB has required that the personal information of the participants is not disclosed. The data that support the findings of this study are available from Armana Ahmed (armana@icddrb.org) to the Research Administration of icddr,b (http://www.icddrb.org/) but restrictions apply to the availability of these data, which were used under license for the current study, and so are not publicly available. Data are however available from the authors upon reasonable request and with permission of Research Administration of icddr,b (http://www.icddrb.org/).
