# Supplementary material for: Effects of probiotic and synbiotic supplementation on ponderal and linear growth in severely malnourished young infants in a randomized clinical trial
Source: Sci Rep. 2023 Feb 1;13:1845. doi: 10.1038/s41598-023-29095-w (PMC9890433; doi:10.1038/s41598-023-29095-w)
Supplement: Supplementary file 1 — Supplementary Information. [file 41598_2023_29095_MOESM1_ESM.docx]

**Supplementary Table:** Composition of F-75 and F-100 used in the study:

|  | F-75 (starter) | F-100 (Catch-up) |
| --- | --- | --- |
|  | Contents per 100ml | |
| Energy (Kcal) | 75 | 100 |
| Protein (g) | 0.9 | 2.9 |
| Lactose (g) | 1.3 | 4.2 |
| Potassium (mmol) | 4.0 | 6.3 |
| Sodium (mmol) | 0.6 | 1.9 |
| Magnesium (mmol) | 0.43 | 0.73 |
| Zinc (mg) | 2.0 | 2.3 |
| Copper (mg) | 0.25 | 0.25 |
| % energy from protein | 5 | 12 |
| % energy from fat | 32 | 53 |
| Osmolality (mOsm/l) | 413 | 419 |
